# Domestic Correspondence

**Published:** 1889-10

**Authors:** 


### Domestic Correspondence.

---

To the Editor:

I have observed that a good deal of attention has been given of late to the subject of crystal gold, and, as I have had a good deal of experience with this preparation of gold, a recital of it may not prove uninteresting to the readers of the International Journal.

It is now nearly twenty-five years since my attention was first directed to Watts's Crystal Gold, and it occurred in this way. Dr. Wetherby, of Boston, was asked to give a clinic at one of the meetings of the American Dental Association, and Dr. B. J. Bing, now of Paris, was the patient. Dr. Wetherby selected for his operation a large compound cavity in an inferior first molar. After preparing the cavity in the usual way, he proceeded to fill the bottom of the cavity with soft foil (rubber dam was not in use in those days), and continued using it until the cavity was half or two-thirds filled. He then finished his filling with Watts's Crystal Gold. I had never seen a more uniform and solid surface than was obtained by that method. Soon after that I purchased some of the gold, and used it as above indicated, and found that the results were most gratifying. I was then led to use it in cavities where no soft-gold foil had been used as a foundation, and the results were equally satisfactory. Many of these operations were made in mouths which I have had an opportunity of examining during all these years past, and the operations thus made look as new or better than those of other preparations of gold made subsequently.

For reasons which I have never been able to satisfactorily explain, I discontinued its use for several years. About 1873 I again began using it, and my opinion of its merits was rather intensified than otherwise; but after a year or two I again abandoned it, and have only occasionally used it until within the past year, when I purchased another lot, and found the same pleasing results attending its use. Last autumn, when comparing methods of practice with Dr. S. G. Perry, of New York, he remarked that he had recently been using a good deal of Watts's Crystal Gold, and with great satisfaction; whereupon we mutually discussed its merits, and mentally analyzed its various properties.

I am convinced that my experience with this gold is in keeping with that of other members of the profession, for inquiry has elicited the information that many have used the gold in question with a great degree of satisfaction, and then, without positive reasons therefor, have abandoned it.

My experience with crystal gold has taught me the following:

That it should be used in moderately small pieces, and with blunt-pointed instruments with shallow serrations.

That in cavities difficult of access, because of the close approximation of adjoining teeth, it is contra-indicated, inasmuch as there is a tendency for it to break into pieces or crumble in the attempt to introduce it.

That when used with care there is no greater liability of imperfect adaptation or discoloration of the tooth than when foil or other preparations of gold are used.

That surface discoloration is not more frequent than by the use of other gold, and that when it does occur it is not because of any adverse conditions inherent in the gold.

That it is specially adapted to large operations where the restoration of contour or great hardness of surface is desired.

That with a given amount of time and labor, better operations can be made with this gold than with any other with which I am familiar.

After having said so much in favor of Watts's Crystal Gold, the reader may infer that I use it to the exclusion of all others, which is not true. I find it most useful in finishing filling, and in cavities easy of access where large or broad-pointed instruments can be used to advantage.

Yours truly, EDWIN T. DARBY.

PHILADELPHIA.

---

TO THE EDITOR:

Just a word in regard to the care of the teeth of the sick. It is surprising to find how few trained nurses give any attention to the teeth of those they have in charge. Everything else is looked after and kept clean but the mouth. The lying-in patient has her hair brushed within a few hours of delivery, but is left for days or weeks without any attention being paid to the mouth; and this, too, in the care of professional nurses under the best physicians. It is astonishing to find how utterly the mouth and teeth are neglected in case of illness, and it behooves every dentist to educate every medical practitioner he comes in contact with in regard to the importance of attention to the teeth of the sick. And something can be done by teaching the laity that they must demand care of the mouth in illness. If germs destroy the teeth, what a good time they must have in the mouth of a typhoid-fever patient; and, in fact, in all cases of adynamic disease. The fever that accompanies it and the diet which has to be given combine to make the mouth a hot-bed of germs, and, no doubt, many cases of carious teeth have arisen from the neglected local conditions, quite as much as from the constitutional depression.

And the comfort to the invalid! If you wish to see a grateful patient, rinse the mouth with some antiseptic solution, after he has been left for days without care! I have had people tell me that nothing done for them in the course of their illness gave them such a feeling of comfort and rest as purifying the mouth.

In extreme cases, where the patient is in a comatose condition, the mouth can be wiped out with a soft cloth wet in the antiseptic solution; but in most cases I have found the ordinary invalids' feeding-cup to answer the purpose nicely. The patient takes the solution into the mouth through the long spout, and, having rinsed thoroughly, closes the lips about the spout and forces the liquid back into the cup,—all done without raising the head from the pillow.

I have no doubt that there are physicians and nurses who attend to this matter, but I also doubt not that they are few and far between. Yours truly,

Edward C. Briggs.

Boston, Mass.

---

To the Editor:

The invisibility of clinics has long been a source of annoyance to operators and a disappointment to observers. At the late meeting of the Massachusetts Dental Society the problem seemed to be solved for once, and I mention it so it may be tried for general application. In giving my description of the operation to be performed I stepped upon a chair, which brought me up sufficiently high to be plainly seen and heard. I then found it practicable to raise the patient sufficiently to accommodate my own elevation. This brought the operation into full view of all standing around, and no one's sight was obstructed, and all got a clear idea of the points made in the operation.

Another operator, giving a descriptive clinic from models, we placed sitting in a chair upon a table, which produced the same desired effect. I suggest, as the outcome of this, a small platform, just large enough for chair and operator, from fifteen inches to twenty inches high. This will bring the patient and operator into full view of all standing around. I hope it may be tried and found to serve the purpose.

It has for many years seemed to me the silk furnished for dentists for ligatures is much coarser than is needed, and causes the patient much pain that might be avoided. I have used with a great deal of satisfaction Eureka embroidery silk, stranded and waxed, a single strand passed twice around the tooth and tied. This will turn up the edge of the rubber dam, and carry it under the gum around the neck of the tooth, with a minimum of compression of the tissue, and causing but little pain. This is worth a trial by those not already familiar with it..

THOMAS HILLEBROWN.

---

TO THE EDITOR:

There appeared in the editorial columns of the *Dental Cosmos* of June, criticisms on remarks I had the honor to make before the New York Odontological Society in March last, to which, with your permission, I would like to reply through your journal.

It seems strange to me that, because some strictures had been made on a commercial house, there should appear, in the editorial columns of what claims to be a professional journal, an attempted vindication of that house. If the answer had come as a communication from that company, all well and good; but it seems to me that in this instance the editor let the commercial side of the question take precedence of the professional, or he considered for the time that his journal was simply a commercial publication devoted exclusively to the interests of that house.

The editor says he "will call attention to but one of the statements reported, although others are equally incorrect." It seems to me he made an invidious distinction, preferring to attack an invited guest of the society, rather than a member of the society whose proceedings they were anxious to keep.

At the time my remarks were made I had every reason, and I still have the same reasons, for believing that in the main the statements were correct. I find one error. I stated that if *I had been rightly informed*, two hundred engines of the Hodge-Weber patent were made and ready to put together; I was not correctly informed. The editor states that "there were not two of them made either in whole or in part." I certainly saw two of them, January 20, 1887, one of which passed into the possession of the S. S. White Company, and the other belongs to and has been used since that time by Dr.

Perry, who made the statement at the meeting where my remarks were made, that the working of it was so satisfactory that he had refused to sell it for five hundred dollars.

What inference could any one draw from the fact that after two and a half years, with two hundred orders in the hands of the original inventors, except that it was not their intention to place it on the market. If two could be made from a pattern, then two hundred could be made from the same pattern. I know that it would take some time to prepare machinery to make them quickly and economically, but they had the model machine for a pattern and Mr. Weber, one of the inventors; but instead of employing him to complete the machine—which Dr. Perry says was then "a more perfect one than any on the market"—to accommodate the two hundred who desired it, they employ him on other things. Is there any wonder that they received criticism for the delay.

I have no quarrel with any man or firm who obtains a patent on the outcome of their brain-work. What I do object to is that any manufacturing house should buy up these patents and neither manufacture them nor allow others to do so, thereby depriving the profession of the benefits to be derived. This is not the only machine by any means that has met this fate.

Any further than this I have no quarrel with the S. S. White Manufacturing Company. I have traded with them for thirty-four years, and in all that time I have found them square-dealing and honorable; so, whatever I have said can result from no ill will I bear them.

G. A. Gerry.

Lowell, Mass., July 25.

---

To the Editor:

In your last number you throw a small rock at the State Board because we were not represented in the meeting at Saratoga. We have felt serious regret each time we have not been represented in national meeting, but several good reasons exist to account for the seeming neglect.

The State Society has taken from the Board those members of college faculties whose interest and pleasure it is to attend the annual meeting of the American Association. Other men have not time or cannot afford the expense of attending, as no fund exists upon which to draw to pay the necessary travelling expenses of representatives.

Then again, the American Association is less popular than it has been with us formerly, and "fails to draw," as theatre men say.

The first meeting of the National Board is called *too soon*,—always at *nine* A.M., the first day of meeting of Association, so that it is often difficult to get there in the forenoon of Tuesday, and always unpleasant to miss that first meeting.

The arrangement to hold the meeting of the State Society one week in advance of the American is almost wholly for the convenience of *city men*, who take one long vacation, and does not so well accommodate others, who are not prepared to use a second week immediately following one devoted to the State Society.

W. E. MAGILL.

ERIE, PENNSYLVANIA.

